# Study design of 'FRIENDS for Life': process and effect evaluation of an indicated school-based prevention programme for childhood anxiety and depression

**DOI:** 10.1186/1471-2458-12-86

**Published:** 2012-01-27

**Authors:** Mia P Kösters, Mai JM Chinapaw, Marieke Zwaanswijk, Marcel F van der Wal, Elisabeth MWJ Utens, Hans M Koot

**Affiliations:** 1 Department of Epidemiology, Documentation and Health Promotion, Public Health Service of Amsterdam, P.O. Box 2200, 1000 CE Amsterdam, The Netherlands; 2 Department of Public and Occupational Health, EMGO Institute for Health and Care Research, VU University Medical Centre, Van der Boechorststraat 7, 1081 BT Amsterdam, The Netherlands; 3 NIVEL, Netherlands Institute for Health Services Research, P.O. Box 1568, 3500 BN Utrecht, The Netherlands; 4 Department of Child and Adolescent Psychiatry/Psychology, Erasmus Medical Centre-Sophia Children's Hospital, P.O. Box 2060 (Kp-2), 3000 CB Rotterdam, The Netherlands; 5 Department of Developmental Psychology, Faculty of Psychology and Education, VU University, Van der Boechorststraat 1, 1081 BT Amsterdam, The Netherlands

**Keywords:** Prevention, School-based intervention, Anxiety, Depression, Children, FRIENDS for Life, Cognitive-behavioural therapy

## Abstract

**Background:**

Anxiety disorders and depression are highly prevalent in children and affect their current and future functioning. 'FRIENDS for Life' is a cognitive-behavioural programme teaching children skills to cope more effectively with feelings of anxiety and depression. Although 'FRIENDS for Life' is increasingly being implemented at Dutch schools, its effectiveness as a preventive intervention in Dutch schools has never been investigated. The aim of the study is to evaluate the effectiveness of 'FRIENDS for Life' as an indicated school-based prevention programme for children with early or mild signs of anxiety or depression.

**Methods/Design:**

This study is a controlled trial with one pre-intervention and three post-intervention measurements (directly after, and 6 and 12 months after the end of the programme). The study sample consists of children aged 10-12 years (grades 6, 7 and 8 of Dutch primary schools), who show symptoms of anxiety or depressive disorder. Data are collected through self-report, teacher report and peer nomination. A process evaluation is conducted to investigate programme integrity (whether the programme has been executed according to protocol) and to evaluate children's and parents' opinions about 'FRIENDS for Life' using online focus groups and interviews.

**Discussion:**

The present study will provide insight into the effectiveness of 'FRIENDS for Life' as an indicated school-based prevention programme for children with early or mild signs of anxiety or depression.

**Trial registration:**

Netherlands Trial Register (NTR): NTR2397

## Background

Anxiety disorders [1] and depression [2] are highly prevalent in children [3]. Anxiety and depression are not only associated with limitations in children's current functioning (e.g., poor social relations and academic performance, low self-esteem) [4], these disorders can also negatively affect children's emotional and social long term development. For example, childhood anxiety and depression are important predictors of psychopathology in adulthood [5,6]. When left untreated, the problems are likely to deteriorate [7]. However, only a minority of children with anxiety and depression receive mental health care for their problems [8-10]. Therefore, early prevention of these disorders is of utmost importance. This may not only have individual benefits with respect to children's current and future wellbeing, but may also serve society as a whole by reducing societal costs related to these problems (e.g., school drop-out, employment problems, health care use, medication) [4].

Since schools offer the opportunity of reaching large groups of children, they are regarded as a suitable setting for the detection, prevention and early treatment of anxiety and depression [11,12]. 'FRIENDS for Life' is a programme that can be used for the prevention and treatment of anxiety and depression in children [13-16]. This cognitive-behavioural programme teaches children skills to cope more effectively with feelings of anxiety and depression and builds emotional resilience, problem-solving abilities and self-confidence.

'FRIENDS for Life' can be used in different forms: 1) as a universal prevention programme for everyone in a specific school population without regard to individual risk factors; 2) as an indicated prevention programme for children with early or mild symptoms of anxiety or depression; or 3) as treatment for children with anxiety disorders. Effectiveness studies conducted in various countries have shown positive results: all three applications of 'FRIENDS for Life' resulted in a decrease in symptoms of anxiety, not only immediately after completion of the programme [17], but also 1-3 years later [18-22]. Due to these positive results, the programme is recommended by the World Health Organization to prevent the development of anxiety disorders in children [23].

'FRIENDS for Life' is increasingly being implemented as a preventive programme at Dutch schools. In Amsterdam, the Public Health Service has been implementing 'FRIENDS for Life' as an indicated preventive programme in primary schools for more than 3 years. However, its effectiveness as preventive programme has never been investigated in the Dutch situation. The fact that 'FRIENDS for Life' has been shown effective as a school-based intervention in Australia [18,20,21], England [22], Scotland [24], South Africa [25], and the United States [19] does not automatically imply its effectiveness and suitability in the Netherlands. Differences in school systems, cultural norms and values, and ethnic and socio-economic characteristics of participating children may result in differences in effectiveness of prevention programmes between countries. In addition, although the effectiveness of the programme has been established in other countries, the effect size varied per study ranging from 0.06 to 2.76 [26].

In this controlled trial, we aim to investigate the effectiveness of 'FRIENDS for Life' as an indicated school-based prevention programme for children aged 10-12 years (grades 6, 7 and 8 of Dutch primary schools) with early or mild symptoms of anxiety and/or depressive disorder. This paper describes the study protocol of the process and effect evaluation of 'FRIENDS for Life'. Children are followed up to 12 months after the intervention. Additionally, we investigate the moderating effect of severity of initial symptoms, gender, age, ethnicity, peer relations, and co-morbid externalising problem behaviour on symptoms of anxiety and depression.

## Methods

### Study design

This study is a controlled trial with one pre-intervention and three post-intervention measurements. The Medical Ethics Committee of VU University Medical Centre approved the study protocol. At least 20 primary schools in Amsterdam planned to participate in 'FRIENDS for Life' in school year 2010/2011 and/or 2011/2012. These schools are asked to participate in the evaluation study. For the control group, other primary schools in the same area in Amsterdam are asked to participate in the study and to participate in the programme afterwards.

### Intervention

'FRIENDS for Life' is a programme teaching children skills to cope more effectively with feelings of anxiety and depression and building emotional resilience. The programme is based on cognitive behavioural therapy (CBT), which has been proven to be effective in the treatment of child anxiety and depression [27]. It encompasses the following techniques: psycho-education, relaxation exercises, exposure, problem-solving skills training, social support training and cognitive restructuring exercises [13]. The letters 'FRIENDS' are the acronym for: ***F**eelings; **R**emember to relax. Have quiet time; **I **can do it! I can try my best!; **E**xplore solutions and Coping Step Plans; **N**ow reward yourself! You've done your best!; **D**on't forget to practise; **S**tay calm for life! *[13,14]. 'FRIENDS for Life' consists of 10 weekly sessions plus 2 booster sessions, one and two months after finishing the programme (see Table 1). The programme also includes two parent sessions during the 10 week-programme.

**Table 1:** Outline of 'FRIENDS for Life' (based on Barrett and Pahl [28])

Session Number	Content of Session - Major Learning Objectives
Session 1	Rapport building and introduction of group participants Establishing group guidelines Normalisation of anxiety and individual differences in anxiety reactions

Session 2	Psycho-education regarding identification of various emotions Introduce the relationship between thoughts and feelings

Session 3	**F**: Feelings (Identifying body signs of anxiety) **R**: Remember to relax. Have a quiet time. (Relaxation activities and identification of pleasant or distracting activities to do when feeling worried or sad)

Session 4	I: I can do it! I can try my best! (Identifying self-talk, introducing helpful green thoughts and unhelpful red thoughts)

Session 5	Attention training (looking for positive aspects in difficult situations) Challenging unhelpful red thoughts **E**: Explore solutions and Coping Step Plans (introducing coping step plans/graded exposure to fear hierarchies, setting goals and breaking problems into small steps)

Session 6	Problem-solving skills (6-Stage Problem-Solving Plan) Coping Role models Social support plans

Session 7	**N**: Now reward yourself! You've done your best!

Session 8	**D**: Don't forget to practise (practising the FRIENDS skills) **S**: Smile! Stay calm for life! (Reflect on ways to cope in difficult situations)

Session 9	Generalising skills of FRIENDS to various difficult situations Teaching others how to use the FRIENDS coping skills

Session 10	Skills for maintenance of the FRIENDS strategies Preparing for minor set-backs that may occur

Booster 1	Review of FRIENDS strategies and preparing for future challenges

Booster 2*	Review of FRIENDS strategies and preparing for future challenges

* This session is not implemented in the present study (see Methods)

In this study, prevention workers of a mental health care organisation deliver the programme in Amsterdam. A 'FRIENDS for Life' group consists of ten children aged 10-12 years (grades 6, 7 and 8) and is run by two prevention workers per group. The intervention programme starts two times a year: at the beginning of the school year and after the Christmas break. Schools choose when they want to start a 'FRIENDS for Life' group, once a year. The way in which the programme is implemented in Amsterdam differs slightly from the original protocol. Firstly, due to low parental attendance in the previous years, only one parent session is organised instead of two during the programme, after session 3 or 4. When the programme is finished, parents are invited for an individual evaluation of the programme with the prevention workers. Because of schools' time constraints, only one booster session is held with the children, one month after finishing the programme. Both child and parent sessions are held at school during school time.

### Measurements

**Figure 1:**
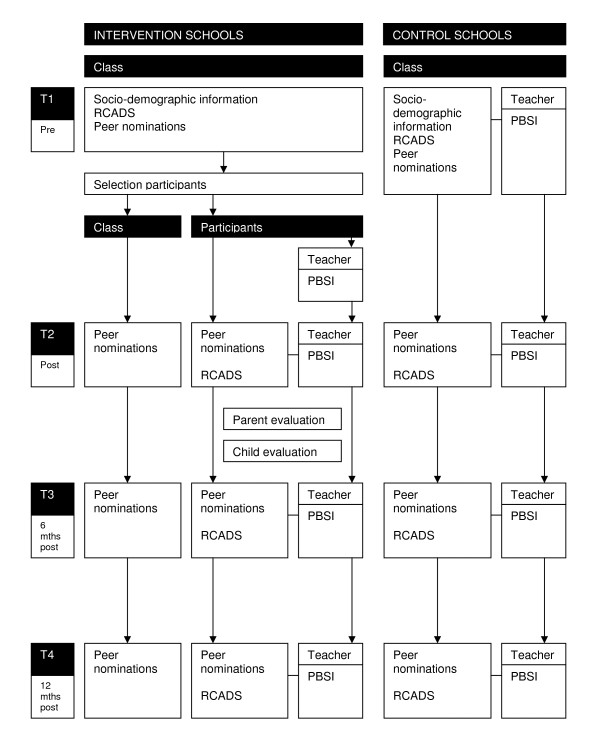
**Flow diagram of the study design**.

The main outcome measures are symptoms of anxiety and depression, externalising problem behaviour and peer rejection at school. Since previous studies found differences between self-report and teacher report of internalising problems, data on symptoms of anxiety and depression are collected through self-report, teacher report and peer nomination [29,30]. In addition to symptoms of anxiety and depression, also behavioural problems and social preference are assessed, because research has shown that internalising and externalising problems tend to co-occur [31,32], and social relationships in the classroom are concurrently and prospectively related to both [33]. Secondary outcome measures are programme integrity, and child and parent evaluations of the programme. For an overview of measures, see Figure 1.

#### Symptoms of anxiety and depression

The Revised Child Anxiety and Depression Scale (RCADS) assesses self-reported symptomatology in children aged 8-18 years, corresponding to DSM-IV criteria for anxiety disorders and depression [34]. It comprises 47 items, which can be combined into six subscales (separation anxiety disorder, social phobia, obsessive compulsive disorder, panic disorder, generalised anxiety disorder, major depressive disorder). Items are for instance *"I worry about things", "I feel sad or empty"*, and *"I feel scared when I have to take a test"*. Children are asked to rate on a 4-point Likert scale how often each item applies to them. Reliability and validity have been shown to be adequate to good [34,35].

#### Peer nominations

Peer nominations of externalising and internalising problem behaviour and peer rejection are obtained with a 7-item scale. Peer nominations of externalising and internalising problems in grades 6, 7 and 8 are obtained by asking all children in a class to nominate all classmates of either sex that fit either of five descriptions: *Starts fights *(conduct problems), *Does not obey school rules *(opposition), *Bullies *(conduct problems), *Is fearful *(anxiety), and *Is sad easily *(depression). Children's nominations for each of these descriptions are divided by the number of children in the class minus 1 (self-nominations are not allowed). The conduct and oppositional problem (*r *= 0.78 in previous studies) nominations are summed to an overall externalising problems score [36]. The anxiety and depression (*r *= 0.57) nominations are summed to an overall internalising problems score (for details, see Van Lier & Koot, 2010) [36].

Social preference scores (SP) are used in this study as a measure of peer rejection. Children are asked to nominate an unlimited number of children in their class who they like most, and who they like least [37]. Children's liked-most and liked-least scores are divided by the number of children in the classroom minus 1 (self-nominations are not allowed). SP scores are computed by subtracting the liked-least score from the liked-most score for each child. Low scores indicate poor acceptance by classmates (peer rejection).

#### Internalising problem behaviour at school

Teacher ratings of internalising problems are obtained by means of the Problem Behaviour at School Interview (PBSI) [38]. The PBSI is a 42-item teacher questionnaire tapping emotional and behavioural problems. The PBSI is presented to the teachers in an interview format, and teachers rate behaviours on a five-point Likert scale, ranging from 0 (*never*) to 4 (*often*). In this study, only the depression and anxiety symptoms scales of the PBSI are used. The depression symptoms scale comprises 7 items. Sample items include "*this child has a lack of energy*", "*this child is indifferent, apathetic or unmotivated*", and "*this child is unhappy or depressed*". In a previous study, Cronbach's alpha ranged from 0.78 to 0.83 across the school years [36]. The anxiety symptoms scale includes 5 items, such as "*this child is nervous or tense*", "*this child worries about many things*", and "*this child is anxious*", with Cronbach's alpha ranging from 0.79 to 0.81 [36].

#### Socio-demographic information

Children are asked to fill in their postal code to assess their socio-economic status. Age, gender, presence of siblings, ethnicity, and history of treatment for internalising and externalising problems are assessed by self-report.

### Procedures

In this study, children in the intervention and control groups are recruited from different schools. Children cannot be included in both intervention and control group.

#### Intervention group

At baseline (T1), all children from grades 6, 7 and 8 of the intervention schools are asked to complete the RCADS and the questions regarding socio-demographic characteristics. Children are also asked to complete the 7-item peer nomination measure. Per school, a group of 10 children is selected (see Inclusion below) to participate in the 'FRIENDS for Life' programme. At the post-intervention measurement (T2), as well as after 6 (T3) and 12 (T4) months, children who participated in the programme are asked to complete the RCADS again. Current teachers of the in 'FRIENDS for Life' participating children are asked to rate internalising problem behaviour with the PBSI at all four time points. The peer nomination measure is completed at all four time points by all children currently in the class to obtain information on children's internalising and externalising problem behaviour and peer rejection. The questionnaires are completed in the classroom during school time. A research assistant is available for additional explanation if needed.

#### Inclusion criteria

In each intervention school, 10 children from grades 6, 7 and 8 with early or mild symptoms of anxiety or depression are selected to participate in 'FRIENDS for Life'. This selection procedure equals current practice for the selection of participants of 'FRIENDS for Life' in Amsterdam.

The selection process takes place in four stages:

1. Children from grades 6, 7 and 8 take a letter home informing their parents about the selection procedure for the programme and evaluation study. For the selection procedure and study participation (i.e., the completion of questionnaires by children), the principle of passive informed consent is used. All children in grades 6, 7 and 8 are asked to fill in the RCADS and a question regarding their motivation/willingness to participate in the intervention.

2. Teachers are informed about the programme and its target group. They are asked to indicate for each child in the class whether he or she fits into the target group of the programme (yes/no). They are asked to indicate why a particular child is selected (shy or withdrawn, (social) anxious, inhibited, is being bullied) or not (shows few or no symptoms of anxiety or depression, bullies other children, is overactive, psychopathological problems already too severe) or other (when none of the above mentioned applies).

3. Children who score highest on the RCADS and/or who are nominated by their teacher and who are willing and motivated to participate in the intervention are eligible for participation in 'FRIENDS for Life'. With this information, the care coordinator of the school (who provides assistance to children with educational and psychosocial needs of children within the Dutch school system) and prevention workers compose a balanced group (regarding gender and age) of 10 children. When more than 10 children are selected, these children can participate in the next scheduled programme or are referred to other relevant programmes.

4. Selected children and their parents receive an invitation for an interview with the prevention workers and an information letter about the study. The prevention workers ask the children and parents which symptoms of anxiety and/or depression they want to address during the programme, and whether the children are motivated enough to participate in the programme. Based on this information prevention workers, children and parents decide together about programme participation. Finally, children and parents are asked if they are willing to participate in the evaluation study. If they consent, parents and children (if older than 11 years) are asked to sign a consent form.

#### Exclusion criteria

Children known by teachers or care coordinators to be referred or already seeking help for a clinical anxiety or depressive disorder, externalising behaviour problems, substantial learning disabilities or a developmental delay are excluded from 'FRIENDS for Life'.

#### Waiting-list control group

The measurements in control schools are scheduled on the same time points as for the intervention group, except that selection for participation in 'FRIENDS for Life' takes place after all data have been collected, i.e. at T4. Because selection takes place afterwards, all children are asked to fill in the RCADS and peer nominations at all time points. Teachers are asked to fill in the PBSI for all children at all time points. After T4, the control schools start a 'FRIENDS for Life' group. At this time, parents/schools of control group children with deviant scores who in the meantime have moved to other primary or secondary schools, will be contacted and advised about possible programmes.

### Sample size calculation

Sample size calculation is based on changes in anxiety and depression assessed by the RCADS. Assuming an alpha of .05, power of 80%, and a 2-sided test, we need 168 participants per group to detect a mean difference of 15-25% on the anxiety and depression scale between the intervention and control group. To be able to perform multilevel analyses, taking into account the clustering within classes and schools and to allow for dropout, a sample size of 202 children per group is required.

### Process evaluation

Programme integrity and children's and parents' evaluations of the programme are investigated in a process evaluation.

#### Programme integrity

Programme integrity refers to the extent to which the programme is implemented as planned [39]. When an intervention is carried out in practice, the implementation is subject to all sorts of threats. This could lead to alterations with respect to the programme protocol. The extent of implementation is likely to affect the effectiveness of an intervention [40]. Therefore, the degree of programme integrity and its influence on programme outcomes are investigated following the model of Dane and Schneider [41] (see Table 2). Data are collected by means of qualitative and quantitative methods:

1. Prevention workers record adherence, participant responsiveness, and exposure in logs.

2. Structured real life observations of the adherence to the programme protocol and the quality of delivery are conducted by trained observers, using a standard checklist [42]. The observers also rate participant responsiveness on a 4-point Likert scale. Notable observations that cannot be rated on the checklist are also recorded. At least two sessions per 'FRIENDS for Life' group will be selected for observation.

**Table 2:** Aspects of programme integrity of 'FRIENDS for Life' (based on Dane and Schneider [41])

Aspect	Definition
Adherence	The extent to which specified program components were delivered as prescribed in programme manuals

Exposure	The number of sessions implemented; the level of attendance and homework finished

Quality of delivery	Therapeutic skills of trainers

Participant responsiveness	Children's response to program sessions, including indicators such as levels of participation and enthusiasm

#### Child evaluation of the programme

A random sample of 40 children who participated in 'FRIENDS for Life' is contacted to participate in online focus groups (OFG) after they finished the programme. OFGs are moderator guided text-based group discussions on the Internet. Previous research has shown that OFGs are usable for this age group [43]. Children receive individual login names and passwords, with which they can anonymously access the OFG website during 1 week. They can log in any time during these days to answer the questions and to comment on the answers of other participants. On five successive days, a question is posted in the OFG. Questions concern children's global satisfaction with the programme and their views on the usefulness and acceptability of specific components of the intervention [44]. The OFGs are moderated by the researchers.

#### Parent evaluation of the programme

A random sample of 40 parents of children who participated in 'FRIENDS for Life' is contacted for a brief structured telephone interview after their children finished the programme to investigate their opinions about and experiences with 'FRIENDS for Life'. If the parent does not master the Dutch language sufficiently, interpreters will translate the interview. Questions concern parents' global satisfaction with the programme and their views on the usefulness and acceptability of specific components of the intervention [44]. Parents are also asked if they have noticed any changes in their child's behaviour during participation in the intervention.

### Statistical analyses

By means of longitudinal multilevel analyses (school, class, individual), we investigate differences in the development of anxiety and depression between intervention and control group, adjusting for baseline values, age, gender and ethnicity. To investigate the potential moderating effect of gender, age, ethnicity, severity of symptoms at the start of the intervention, peer relations, and co-morbid externalising problem behaviour, an interaction term between group and the respective moderator will be added to the model. Because of the limited sample size, these subgroup analyses will be performed on an exploratory basis only.

For the process evaluation, descriptive statistics of the logs and observations are calculated. Data of the online focus groups and the interviews will be analysed using text analysis software.

## Discussion

This study evaluates the effectiveness and implementation of 'FRIENDS for Life' as an indicated prevention programme. Application of an intervention in the school setting provides the opportunity to reach large numbers of children in a relatively safe and non-stigmatising environment. Easily accessible prevention can be an important strategy to reduce the high prevalence of anxiety and depression in children. However, policy makers in the Netherlands are hesitant to implement prevention programmes on a large scale because of poor scientific evidence of these programmes. Therefore, the present study aims to add to evidence-based prevention of anxiety and depression in children.

### Strengths and limitations

This study has a number of strengths. Firstly, we evaluate current practice since the Public Health Service of Amsterdam has been implementing 'FRIENDS for Life' for more than 3 years. This experience with the implementation of 'FRIENDS for Life' resulted in a feasible study protocol enhancing the response rates in the study.

A second strength is that this study not only evaluates the effectiveness of 'FRIENDS for Life' for the total sample of participating children, but also investigates whether the programme has more benefits for specific groups of children than for others. The pupils of primary schools in Amsterdam vary widely in ethnic background and socio-economic status, which guarantees a diverse study population. Based on insights from subgroup analyses, 'FRIENDS for Life' can be targeted more specifically to certain groups of children, and alternatives can be sought for groups of children for whom the programme may be less effective. These insights contribute to a better tailored and cost-efficient implementation of 'FRIENDS for Life'.

A third strength is the study design, in which short-term effects as well as long-term effects of the programme are evaluated, and which includes multiple informants (i.e., children, parents, teachers and peers). Previous research has shown differences between children and teachers in reports of the occurrence of anxiety. Dadds et al. found that teachers' nominations and self-report led to nearly the same rates of detection of anxiety problems (up to 75%), but only 9% of the children were selected by both methods [29], which favours parallel use of both measures.

A limitation of this study is the non-randomised design. Because schools already planned to participate in 'FRIENDS for Life' and started the implementation, randomisation was not possible. This has led to a controlled design comparing children from intervention schools and matching control schools.

A second limitation is that one parent and one booster sessions are implemented instead of two. However, changes in the original 'Friends for Life' protocol seem to happen more often than not. Other effect evaluation studies do not mention the implementation of booster [24,45-47] and/or parent [46,47] sessions or excluded booster [25] and/or parent [25,45] sessions.

## Conclusion

The present study will provide insight into the implementation and effectiveness of 'FRIENDS for Life' as an indicated school-based prevention programme for children with early or mild signs of anxiety or depression.

## Competing interests

The Department of Child and Adolescent Psychiatry/Psychology at Erasmus Medical Centre is publisher of the Dutch 'FRIENDS for Life' protocol, from which Dr. E. Utens receives remuneration. The other authors declare that they have no competing interests.

## Authors' contributions

All authors participated in the design of the study. MK generated the first draft of this manuscript based on the original study protocol and is responsible for the data collection and analysis. MC, MZ, and HK are the principal investigators of this study and revised the manuscript. All authors contributed to the further writing of the manuscript. All authors read and approved the final manuscript.

## Pre-publication history

The pre-publication history for this paper can be accessed here:

http://www.biomedcentral.com/1471-2458/12/86/prepub
